# Determination of a strength index for upper body local endurance strength in sedentary individuals: a cross sectional analysis

**DOI:** 10.1186/s40064-015-1539-9

**Published:** 2015-11-25

**Authors:** Ewan Thomas, Antonino Bianco, Marianna Bellafiore, Giuseppe Battaglia, Antonio Paoli, Antonio Palma

**Affiliations:** Sport and Exercise Sciences Research Unit, University of Palermo, Via Giovanni Pascoli, 6, 90144 Palermo, Italy; Department of Biomedical Science, University of Padova, Via Manzolo 3, 35131 Padua, Italy

**Keywords:** Posture, Strength, Normative values, Sedentary

## Abstract

A range of balance between flexor and extensor muscles is fundamental in order to prevent pathologies caused by bad postures or to ensure health of the joint as a measure of prevention of overtraining in specific muscle groups. Therefore, the aim of this study is to examine the ratio between “pulling” and “pushing” strength in sedentary individuals. 212 healthy participants, of both genders (139 male and 73 female; age 32 ± 13.3 years, weight 70.2 ± 14.1 kg, height 173 ± 9 cm) were retained for investigation. Strength was assessed through a new methodology: Pulling through a lat-pulldown test while pushing strength through a chest-press test. Both tests were performed to exhaustion with an overload of 30 % of each participants bodyweight. Such method aims to prevent excessive overloads in sedentary individuals. Pearson’s correlations and a t test to assess differences were analyzed. Subsequently, the ratio for both genders of pulling and pushing local endurance strength was assessed by means. A mean number of 57 repetitions was shown with the lat-pulldown while 34 repetition with the chest press. A correlation of 0.42 has been found between the number of repetitions of the two tests. A significant difference (p < 0.001) was found between such performances. No correlation was found between the strength measures and the anthropometric parameters of the participants. The lat machine to chest press ratio was 1.36:1 for male while 2.69:1 for female. The results indicate that sedentary participants have higher pulling rather than pushing local endurance strength. Such ratio should be considered as a normative value when starting to perform exercise protocols. Resistance training should be performed in order to improve strength measures of the weaker muscles and reduce such ratio.

## Background

During the last decade a vast number of studies have investigated upon muscle balance regarding the different strength outcomes between the hemisomas or the different ratios amongst agonist and antagonist muscles crossing a specific joint (Negrete et al. 2010; Behm et al. 2002; Kang et al. 2014). Such balance, emerges to be an outcome measure from a strength ratio between agonist and antagonist muscles, where the value of one represents an equal strength measure between the “two” muscle districts (Noffal 2003; Bogdanis and Kalapotharakos 2015; Hadzic et al. 2014; Baker and Newton 2004). Baker and Newton, have also posited that a certain balance between antagonist muscles is needed and have investigated such measure in professional athletes (Baker and Newton 2004). In general an optimal ratio gives indication on the state of health of a joint, for example higher ratios imply higher strength of agonist or antagonist muscle that may lead to malpositions or altered movement patterns (Borloz et al. 2012). In addition, if such ratio is applied to postural muscles, an imbalance can lead to gait disorders that inevitably reduce quality of life. Muscle imbalances have been defined as faulty relationships between the antagonist and the agonist muscles that will result with an effect upon the joint they cross (Sahrmann 2002). It is known that an antagonist muscle acts as a joint stabilizer during movement or performance (Bogdanis and Kalapotharakos 2015). An increased level of strength of a muscle compared to its antagonist, therefore, can lead to an increased movement speed or greater peak torque production towards one direction that over time could result in a strain of the weakest muscle (Barlow et al. 2002; Jaric et al. 1995; Bogdanis and Kalapotharakos 2015). Several authors have also reported that overtraining of a specific muscle group, due to the kind of practiced sport or for aesthetic purposes, can lead to injuries or musculoskeletal pathologies (Kolber et al. 2009; Murgia 2013). Overhead sports, for example, such as baseball, or volleyball and sports such as wrestling and gymnastics in which the athletes are pressing or pulling against an opponent or apparatus (Escamilla and Andrews 2009), may predispose athletes to an increased injury incidence of specific muscle groups due to muscle imbalances and overuse, or as posited by Nikolaidis P. a misbalance combined with sport specific training may lead to postural disorders (Nikolaidis 2010). Wang and Cochrane at this purpose report that an imbalance in the strength of rotator muscles plays an important role in shoulder injuries in elite volleyball players (Wang and Cochrane 2001).

Various authors, have theorized that an optimal ratio (defining optimal a value of 1) between the strength of antagonist muscles is a key factor for injury prevention and to elicit performance (Baker and Newton 2004; Negrete et al. 2013). Isokinetic testing has been used at this purpose to investigate muscle imbalances. Baker and Newton in 2004 have evaluated highly skilled athletes, assessing upper body maximal strength of both “pushing” and “pulling” strength through a 1RM bench press and 1RM pull up, respectively. Their outcomes revealed that in such population a ratio of approximately 97 % (bench/pull × 100) between the muscle groups was displayed with a high correlation between tests (r = 0.81). These data indicate that physical activity to an extreme, in this case professional rugby, reduces the ratio between the muscle groups.

Another author (Negrete et al. 2013) has also investigated the existing ratio between pulling and pushing strength. The cohort taken in consideration comprised healthy recreational active individuals. Such sample compared to the Baker and Newton’s one, had a general lower level of physical conditioning. Two separate tests were used (a timed push up test and a modified pull up test performed to exhaustion) and the ratio between pulling and pushing strength was ranging between 1.5 and 2.7, according to gender. Such ratio highlights that active individuals have in general stronger muscles involved in pushing rather than pulling.

It is still unclear which are the key components of these strength variations, if the different assessment methodologies or the different populations, and little is also known about the strength relation of sedentary individuals regarding agonist and antagonist muscles of the upper body.

Therefore, the aim of this study is to understand the strength of “pulling” and “pushing” muscle groups of the upper body in sedentary individuals and to create a ratio that could be used as a normative value for similar cohorts. The use of a new, easy methodology using two different field based fitness tests will be administered.

## Methods

### Participants

Two-hundred and twelve healthy sedentary participants (age 32.5 ± 13.3 years, weight 70.3 ± 14.1 kg, height 172.7 ± 8.6 cm) were enrolled for investigation, these were 139 male and 73 female (Table 1). The participants were enrolled in a commercial gym and were tested within the first week after subscription. The exclusion criteria were: (1) pathologies or any physical injury; (2) regular consumption of medications (regular was defined as at least once a week); (3) resistance trained or endurance trained participants with more than two consecutive weeks of conditioning.

**Table 1:** Anthropometric characteristics of participants

	Male (139)	Female (73)
Age	31.9 ± 13.9	33.6 ± 11.8
Height (cm)	176.8 ± 6.7	165.0 ± 5.8
Weight (kg)	76.7 ± 12.6	58.1 ± 6.9

The values are expressed as means ± SD

The principles of the Italian data protection act (196/2003) were observed. All participants were instructed about procedures, risks and benefits of the study. Informed consent was also provided and all procedures were approved by the ethics committee of the University of Palermo (Sport and Exercise Science Research Unit). The study was performed in compliance with the Helsinki declaration.

Anthropometric measures were assessed in order to apply individualized protocols. Body weight was assessed to the nearest 100 g using a scale (SECA 709, Hamburg, Germany) and height to the nearest mm using a wall-stadiometer (SECA 220, Hamburg, Germany).

### Testing procedures

Each subject had to perform two endurance strength tests: The lat-pulldown (LPD) test and the chest-press (CP) test. The first for pulling local endurance strength and the latter for pushing local endurance strength. Each test was performed to exhaustion using a work load of 30 % of the body weight of each participant (such weight was used in order to avoid injuries with higher workloads). The sensitivity of the workload was ±1 kg. As seen by several authors (Sentija et al. 2009; Zoeller et al. 2005; Flouris et al. 2006; Mayhew et al. 1995; Aguiar et al. 2015) there is a relationship between anaerobic performance or strength and muscular endurance, for such reason the strength assessment was carried out through two muscular endurance tests, where the use of maximal tests could be dangerous in this cohort.

Each participant had to perform a general warm-up, that consisted of 5 min on a treadmill at a preferred walking speed. The participants weren’t allowed to run, to avoid fatigue prior the testing.

The protocol of the LPD test consisted for each participant in sitting on a lat pull down machine with the knees at 90° compared to the thighs and the body perpendicular to the floor. From this starting position the participant was instructed to grab the pull-bar positioned above the head, raise the arms, having these completely extended and to position them just shoulder width apart on the bar. When the participant was ready, the investigator instructed the participant to pull the bar towards the chest without any involvement of the trunk flexors nor extensor muscles (the repetitions ware performed using only the movement of the arms). The test ended when the participant wasn’t able to perform any more repetitions (to exhaustion) as described in the protocol or when the form became incorrect.

The protocol of the CP consisted in seating the participant in the chest press machine in a comfortable position, with the back fully compliant to the machine and grabbing the handles of the CP from the seated position, having both elbows flexed at 90°. From this position each participant was instructed to push the handles till the arms ware completely extended. This movement had to be performed without any involvement of the trunk flexors nor extensor muscles. The test finished when the participants were not able to perform any more repetitions (to exhaustion) as described by the above protocol or when the form became incorrect.

The protocols were shown by the investigator to each participant just after the verbal instructions as above described.

The tests were randomized with a rest of 10 min between each other and were supervised and administered by the same investigator.

### Statistical analysis

All values were expressed as means ± standard deviations and statistical significance was accepted at p < 0.05 using a two tailed, unpaired, t-test for independent samples. Correlations were assessed through the Pearson’s coefficient. Subsequently, a pull–push ratio was calculated dividing the number or repetitions of the LPD test with the number of repetitions of the CP test (LPD/CP). Statistical analyses were performed through “Statistica 10.0 for Windows (Statsoft Inc., Tulsa, OK, USA)”.

## Results

A mean number of 57 ± 27 repetitions was shown for the LPD test while a mean number of 34 ± 18 repetitions was shown for the CP test (Table 2). When stratified by gender a mean number of 56 ± 26 reps for men and 58 ± 28 for women were shown in the LPD test; whereas a mean number of 41 ± 17 reps for men and 22 ± 10 for women were shown in the CP test. Notwithstanding the differences between the overloads used between male and female (p < 0.001), for both LPD and CP test, no significant differences were observed when comparing the number of repetitions between genders for the LPD test (p = 0.67) whereas the means for the CP tests differed significantly (p < 0.001). These results didn’t show correlations with the anthropometric parameters (age vs. LPD-reps r = 0.02; age vs. CP r = 0.08; weight vs. LPD r = −0.16; weight vs. CP r = 0.39) and a low correlation was also shown between the repetitions of the two tests (r = 0.42). The results indicate that sedentary participants have higher pulling rather than pushing strength and lack of symmetry between antagonist muscles (Table 2). The LPD test to CP test ratio was 1.36 for male while 2.69 for female, indicating a relatively higher muscle strength balance for sedentary men compared to women.

**Table 2:** Number of repetitions and ratio of participants

	Male (139)	Female (73)
Lat pull-down test (reps)	56 ± 26	58 ± 28
Chest-test (reps)	41 ± 17	22 ± 10
Ratio lat:chest	1.36	2.69

The values are expressed as means ± SD

## Discussion

The aim of the study was to examine the strength ratio between antagonist muscles in sedentary individuals in the upper body and provide normative values for such population. The main results seem to suggest that in sedentary individuals a higher strength is present in the pulling musculature rather than the pushing one [The main muscles involved in the chest press exercise are the pectoralis major, the anterior deltoid, the triceps brachii and the rectus abdominis whereas the main muscles involved in the LPD exercise are the latissimus dorsi, the trapezius, the biceps brachii, the posterior deltoid and the rectus abdominis (Uribe et al. 2010; Campbell et al. 2013; Sperandei et al. 2009; Doma et al. 2013)]. As an outcome of the strength measures the ratio is higher in women. Interestingly, the results here provided seem to have an opposite trend to those found in active individuals (Negrete et al. 2013) and those provided in 2004 with highly skilled athletes (Baker and Newton 2004). There seems to be a relation between the level of practiced resistance exercise and the calculated ratio. However, a comparison is difficult to assess due to the different methodological approaches used in the studies. Negrete et al. uses a standard and modified push up test and a modified pull up test (both having the body on a horizontal plane), Baker and Newton use the 1RM bench press test and the pull up test (the first having the body on a horizontal plane and the latter on a vertical plane); while the tests administered in our study were the CP test and LPD test (The first assessed on a transversal plane and the latter on a vertical plane). Notwithstanding the different planes of assessment, all the pushing and pulling tests, assess strength in the same muscle groups, respectively [the bench press and the push ups have as main target the Pectoralis major, the anterior deltoid and the triceps brachii (Lauver et al. 2015; Calatayud et al. 2014); the pull ups have as main target the latissimus dorsi, and the biceps brachii (Youdas et al. 2010)] and are somehow similar. Other consideration that may be taken into account could be the overall different muscle abilities assessed from the different tests. For example the 1RM bench press assesses maximal upper body pushing strength while the push-ups and the CP test assess maximal local endurance upper body strength. The 1RM bench press is the gold standard for upper body strength evaluation (Delextrat and Cohen 2008; Headley et al. 2011; Mayhew et al. 2002) and is widely considered as the most accurate amongst the upper body evaluation tests; the modified push up test adopted by Negrete et al. had been previously tested for reliability by the same authors (Negrete et al. 2010) and it has also been shown to be an effective method to measure upper body strength. Interestingly the outcomes that we provide are not related neither to age or weight (even though the main innovation of our methodology was the normalization set upon the weight of each participant), indicating that the adopted tests are providing reliable results based on the pulling or pushing strength and not influenced by the anthropometric parameters of the participants. If the anthropometric parameters of each participant doesn’t influence the results, and the workload is based upon each one’s weight, and the LPD and CP tests have as main target the same muscle groups seen with the other tests, the differences in the ratios could be explained by the different grade of strength conditioning of each sample, underlining that higher grades of conditioning lead to a ratio closer to 1.

An important consideration, has to be taken into account when generalizing amongst strength conditioning. As found by Baker and Newton, in elite sports, ratios of approximately 1 can be found in those who exercise both pulling and pushing movements at the same grade (gymnastics, weight-lifting, combat sports, ext.), while sports like rowing, swimming or kayaking will be more likely to express higher pulling ratios while sports like boxing or tennis higher pushing ratios (Baker and Newton 2004). In our untrained sample the strength is mainly determined by the activities of daily living, hence a large heterogeneity was expected. Notwithstanding our expectations a consistent outcome for the pulling measures was shown across genders. While a significant difference was shown for the pushing measures between the two groups. A rational amongst the large standard deviation of the pushing measures still needs to be understood making this large deviation the main limit of our study. Such large deviation (±18 repetitions) may also explain why the two tests don’t show correlation. Our ratio is not as selective as that provided for elithe athletes and may be used for comparisons in other untrained populations or similar categories.

## Conclusion

Strength measures in sedentary individuals show a high heterogeneity, thus a normative value could give the means for comparisons. Resistance training in pulling and pushing musculature would lead to reduce the ratio and theoretically balance the strength outcomes. Progressed injuries or traumas must be considered before the administration of the tests in order to avoid biased results.
